# Challenges in managing elderly people with diabetes in primary care settings in Norway

**DOI:** 10.3109/02813432.2013.854445

**Published:** 2013-12

**Authors:** Marit Graue, Trisha Dunning, Marie Fjelde Hausken, Berit Rokne

**Affiliations:** ^1^Centre for Evidence-Based Practice, Bergen University College, Bergen, Norway; ^2^Centre for Nursing and Allied Health Research, Deakin University and Barwon Health, Geelong, Australia; ^3^Department of Medicine, Section of Endocrinology, Stavanger University Hospital Stavanger, Norway; ^4^Department of Global Health and Primary Health Care, University of Bergen, Bergen, Norway

**Keywords:** Chronic illness, diabetes, elderly people, focus groups, general practice, home-based services, Norway, nursing homes, qualitative research

## Abstract

**Objective:**

To explore the experiences and clinical challenges that nurses and nursing assistants face when providing high-quality diabetes-specific management and care for elderly people with diabetes in primary care settings.

**Design:**

Focus-group interviews.

**Subjects and setting:**

Sixteen health care professionals: 12 registered nurses and four nursing assistants from nursing homes (10), district nursing service (5), and a service unit (1) were recruited by municipal managers who had local knowledge and knew the workforce. All the participants were women aged 32–59 years with clinical experience ranging from 1.5 to 38 years.

**Results:**

Content analysis revealed a discrepancy between the level of expertise which the participants described as important to delivering high-quality care and their capacity to deliver such care. The discrepancy was due to lack of availability and access to current information, limited ongoing support, lack of cohesion among health care professionals, and limited confidence and autonomy. Challenges to delivering high-quality care included complex, difficult patient situations and lack of confidence to make decisions founded on evidence-based guidelines.

**Conclusion:**

Participants lacked confidence and autonomy to manage elderly people with diabetes in municipal care settings. Lack of information, support, and professional cohesion made the role challenging.

This study enhances understanding concerning the clinical challenges nurses and nursing assistants face when providing high-quality diabetes care for elderly people.

- It is essential that nurses and nursing assistants in primary care have access to current information and ongoing support.
- They need enhanced professional authority and confidence to manage challenging clinical situations and make appropriate care decisions.
- Reflective practice forums and targeted education and training programmes are needed in primary care settings.
- Specialist health care services and general practitioners with expertise in managing diabetes have a pivotal role in developing programmes that enhance the skills and competence of nurses and nursing assistants.

## Introduction

The dramatic increase in the prevalence of diabetes worldwide threatens the health and quality of life of millions of people and represents a major challenge to already burdened health care services in providing high-quality diabetes management [1,2]. Many elderly people have several comorbidities and complex care needs, take multiple medicines, are vulnerable to isolation and depression, and are at risk of adverse events such as falls and medicine mismanagement [3,4].

Nursing home residents in the United Kingdom have an average of four comorbid conditions in addition to diabetes and have significant care needs and deficiencies [5,6]. Elderly people living at home or in nursing homes in the United States receive inadequate medical care, and the care of elderly people with diabetes in extended-care facilities does not meet American Diabetes Association (ADA) guidelines [7,8]. In addition, care of elderly people is inadequate in the Nordic countries [9,10]. For example, less than 20% of the people with type 2 diabetes in Norway achieve the recommended treatment goals in primary care [9], and diabetes care in Sweden's home nursing services is inadequately documented [10].

In the future, more care for elderly people will be transferred from specialist to primary care in the Norwegian health care services [11]. Patients who live at home receive follow-up care from the district nursing service. Several studies show that nurses’ knowledge concerning diabetes and its care is inadequate [3,12]. Further, evidence indicates that diabetes training for community-based health care professionals significantly improves the metabolic control and quality of life of people with diabetes [13] and that good diabetes management can prevent or delay the development of diabetes-related complications [14].

Norway's new primary care reforms and policies represent an opportunity to further explore the determinants of high-quality care for elderly people with diabetes and the knowledge and competence nurses and nursing assistants need to care for elderly people with diabetes. The current study therefore aimed to explore the experiences and clinical challenges primary care nurses and nursing assistants face when providing high-quality diabetes care for elderly people.

## Material and methods

### Participants and recruitment strategy

We carried out the study in a municipality in southern Norway with 60 000 inhabitants. Some 17% of nursing home residents in the region have diagnosed diabetes [15]. The municipality has five zones: each has a base for coordinating home-based services and nursing home care in the nine municipal nursing homes. We aimed to recruit nurses and nursing assistants from all five municipal zones.

The municipality's nursing management (nurse leaders in charge) invited 21 nurses and nursing assistants to participate in the study, and 16 (12 registered nurses and four nursing assistants) participated. Some of the participants were novice practitioners whereas others had previously been interested in diabetes care. All 12 registered nurses had bachelor's degrees in nursing, and one had specialist training in geriatrics and psychiatry. The four nursing assistants had undertaken the formal Norwegian Nursing Aide training (two-semester programme). All participants were women, aged 32–59 years with clinical experience ranging between 1.5 and 38 years. Ten participants worked in nursing homes, five in the district nursing service, and one in a serviced unit. Fewer frail elderly people live in services units than in nursing homes. Eight participants worked full time. Participants’ clinical experience caring for people with diabetes ranged from five to more than 20 years.

The participants were responsible for providing care to men and women aged 62–97 years with type 1 or type 2 diabetes. Some elderly people also had dementia, cardiovascular disease, chronic obstructive pulmonary disease, stroke, kidney problems, Parkinson's disease, schizophrenia, depression, or were overweight.

### Data collection

We conducted focus-group interviews because they are interactive and participants can share and compare experiences, which enables shared opinions to emerge and generates deep insights into complex issues that enables patterns and trends to be identified within and among groups [16,17]. Four focus groups with respectively four, five, four, and three participants were organized. We developed a semi-structured interview guide to guide the discussion based on the study aims.

The researchers are all female registered nurses: three researchers and one charge nurse. MG, BR, and TD are experienced researchers and have extensive knowledge of diabetes; MFH has first-hand knowledge of both primary and specialist diabetes care. One of the researchers moderated the groups, which were audiotaped. In addition, notes were taken during the discussion to capture non-verbal language and clarify indistinct audiotaped information. Following each focus group, the discussion themes were summarized for the participants to clarify and correct any misunderstandings. We transcribed the audiotapes verbatim after each focus group and checked the transcripts for accuracy. All audiotapes were listened to and cross-checked with the transcripts and the notes.

### Data analysis

We used Kvale's [18] approach to qualitative content analysis to analyse the data and find meaning and categories, condense the categories, and interpret the meaning. We read through the transcribed data once to gain a general understanding of the text. The transcriptions were then reread and categories were formed on the basis of the interview guide. During this process, the text was searched for units of meaning that corresponded to the categories. We condensed them and placed them in appropriate categories. We sorted the categories and their units of meaning again because some units were originally placed in more than one category.

## Results

The participants experienced numerous clinical challenges providing care for elderly people with diabetes. Three main themes emerged: the availability and accessibility of information and support, professional cohesion, and confidence and autonomy.

### Availability and accessibility of information and support

The most common theme emerging in all groups was that having diabetes-related knowledge and information and imparting it to patients, relatives, and colleagues are fundamental to high-quality care. Nurses’ knowledge and proficiency in diabetes care, and the importance of nurses’ confidence in their professional ability were exemplified by the following quote:

You must at least have a lot of knowledge and information yourself … the more you know yourself the more you can observe, maybe … the more you know how to supervise [staff, people with diabetes and relatives], maybe.

Nurses and nurse assistants in primary care settings depended on various types of support from colleagues with specific clinical knowledge of diabetes to interpret and act on their clinical observations. In particular, participants highlighted the importance of good communication with general practitioners to determine their confidence and autonomy to make clinical decisions. This, in turn depended on their diabetes-related knowledge and competence:

We have to find out what to discuss with the doctor and what we can do ourselves … and whether it is good enough for … what is best for the patient. And for that you have to have knowledge … and if we don't know what to evaluate, it most likely won't be discussed with the doctor, and the patient will miss out [on better treatment].

Lack of diabetes-specific knowledge on which to make sound clinical decisions based on clinical observations and patient assessments was challenging for the participants. Lack of confidence in their ability to manage complex patient situations was particularly demanding because many doctors providing home-based diabetes services gave inconsistent advice. In addition, participants often felt alone and unsupported when doctors indicated they were uncertain about standards of diabetes care and were reluctant to make medical decisions. The participants found general practitioners’ uncertainty especially confusing because they are responsible for prescribing insulin and managing unstable blood glucose levels. One participant said:

You sit there and feel completely alone with an insulin problem.

Further, nurses described a range of challenges in observing and detecting changes in the patients’ situation and/or needs and their ability to interpret changes and to manage the change. The participants wanted guidance on exactly what signs and symptoms to look for from specialist diabetes clinicians. One participant said:

What I would like … there is a palliative team at the [hospital] that is very good to have and that you can call … I would like to have [the specialists] come [to the users] and see how their blood glucose is and be guided *exactly* in relation to each user.

### Professional cohesion

Participants felt professional cohesion was often lacking although many nurses and nursing assistants worked the same shifts in district nursing services and in nursing homes. Participants reported inadequate teamwork and inconsistency delivering diabetes care because the aims and standards of care were not consistent. For example, there are:

… so many groups and so many shifts, and everyone has different opinions about something.

Improved team communication would encourage collaboration and facilitate consensus about how to implement evidence-based guidelines. While reflecting on how a team approach improved the leg ulcers of one elderly woman with diabetes, one participant said:

… we have worked well together in relation to [preventing and treating foot ulcers] and … the whole personnel group has had a common understanding about it and what we should look for in relation to minor signs of developing ulcers … we have become good at preventing ulcers.

Further, participants indicated that communication was inadequate within and among the professional disciplines and there were deficiencies in working together. One participant said:

[what we do] is somewhat in bits and pieces … we don't always follow the interventions that we set … it is so important that everyone work in the same way, that we be a team: otherwise the intervention is worth nothing.

### Lack of professional confidence and autonomy

The participants stated that they require adequate training and support and enhanced professional competence and confidence to have autonomy to make care decisions. They indicated that competence in specialties such as diabetes has become increasingly critical to enable primary care nurses and nursing assistants to observe, evaluate, and act on the complex needs of the growing ageing population. However, as one participant reported:

… courses are often sporadic … “you and you can take a course”, there is no teaching plan at the nursing home….

Moreover, prioritizing and balancing care delivery with the documentation required to comply with regulations and standards of care are challenging. In addition, work routines were lacking or were not followed or evaluated:

… when they [nurses] are at work, they have so much to do that keeping nursing plans and interventions updated falls away….

There appears to be a need to provide nurses in primary care settings with enhanced professional authority and confidence to enable them to take the lead in delivering high-quality diabetes care. Heavy workloads and limited time to complete tasks because of understaffing hamper nurses’ confidence and autonomy to deliver diabetes care.

Dilemmas related to lack of capacity to provide high-quality care also correspond to organizational challenges faced by health care services. Many services do not have enough nurses employed which means many nursing assistants work outside their scope of practice and competence, which puts patients at risk:

… [assistants] are given a responsibility that I wouldn't have believed it was legal to give them in relation to going to the units and distributing tablets and doing considerable work that I feel a long way down in my gut.

## Discussion

### Principal findings

The findings suggest there is a discrepancy between the knowledge and expertise participants described as being important for delivering high-quality care for elderly people with diabetes. Their actual capacity to deliver quality of care was compromised by many challenges of daily clinical practice, including lack of access to current information, limited professional support, and inadequate professional cohesion. In addition, participants lacked confidence and autonomy. Poor communication among care providers and within disciplines, the discrepancy between the level of professional expertise participants described as being important to the delivery of diabetes management, and the actual care they were competent to deliver was compromised by several challenges. These included staffing and roster issues that need to be addressed at service and clinical levels.

Participants described challenges in relation to the working environment and the organization of health care. Consequently, participants often felt alone and unsupported by experienced clinicians in diabetes care, which they construe as a lack of a cohesive professional approach. In the future, efforts should focus on establishing systems such that specialist health care contributes to developing expertise in a reinforced municipal health service in primary care settings in Norway.

### Limitations and strengths

One limitation was participant recruitment. We asked municipal managers to help with recruitment because they knew the workforce and had established networks. The participants responded to the request from their leaders to participate in the study concerning the challenges in providing high-quality care. Thus, they may have been more receptive to sharing their understanding of the inadequacies than nurses and nurse assistants who did not participate. Also, power differences within focus groups can affect group processes. Some participants knew each other quite well because they worked together in the same zone, whereas others met for the first time. Some participants may not disclose important information or participate less fully or openly because of experiencing less power, and participants from the same population might provide different information. Each focus group comprised only 3–4 participants. Interaction among participants can be both the key to success and the root of failure in focus groups. Small groups may generate excessive pressure to speak; large groups might inhibit interaction. Krueger & Casey [16] suggested focus groups ideally consist of 6–10 participants. Our choice to conduct smaller groups points to some of the factors related to teamwork such as understanding roles and responsibilities, which might not have emerged in larger groups. Likewise, more examples of doctor–nurse collaboration and team communication that worked may have been identified if we had included members of other professions, who might have different opinions from those of the study participants. In addition, all care personnel in the municipality were women, and thus only women participated.

One strength was including participants providing community care from all five municipal zones and those working in nursing homes and home-based services. Strategies to strengthen reliability include using a semi-structured interview guide carefully designed by both experienced diabetes nurse researchers and a clinical charge nurse. The guide elicited the required information to meet the study aim.

### Discussion of key findings

Participants stated that many patients had comorbid health conditions, which is usual among elderly people with diabetes [19]. Multi-morbidity increases markedly with age [20], increases the complexity of care required, and significantly increases the risk of polypharmacy and adverse events such as falls and pain. Further, participants’ comments and the way they expressed themselves showed they often felt uncertain about what to do, especially when the primary care doctor appeared to have little diabetes knowledge. The study suggests that primary care nurses and nursing assistants lack professional support, particularly from general practitioners. Our findings are similar to those of Cytryn et al., who reported that primary care practitioners had knowledge deficits and lacked confidence, particularly in prescribing insulin, managing the complexities of diabetes, and competence in using diagnostic and treatment guidelines to plan and monitor care [21]. Improved collaboration depends on developing professional relationships among general practitioners, practice staff, and allied health professionals [22]. Factors such as lack of face-to-face interaction and poor understanding of the roles of other professions’ competences might limit teamwork and professional relationships.

Participants in the present study described a work environment that appears to be fragmented. The work environment was characterized by many work shifts consisting of many staff members, lack of consensus on treatment, and lack of cohesion about how to implement diabetes care. Inadequate teamwork and poor management can affect the physical and psychological well-being of elderly people with diabetes. The ADA Standards of Medical Care in Diabetes [23] indicate that a fragmented, inadequately designed delivery system contributes to suboptimal chronic disease care. Personnel must have access to current information and resources and ongoing support to increase their confidence and autonomy to make appropriate care decisions founded on evidence-based guidelines. People with chronic illness, especially elderly people with diabetes living at home or in nursing homes, do not receive optimal care [7,8,24,25].

There is sound evidence that a “team” approach to chronic illness care, where the team has expertise in managing chronic illnesses using evidence-based guidelines, improves patient satisfaction, health care professionals’ adherence to guidelines, clinical outcomes, and health status, and reduces the utilization of health services [26,27]. More specifically, well-coordinated primary care teams that have clearly defined and explicitly delegated roles, as well as the necessary competence, and who meet regularly can deliver high-quality care to people with chronic illness [26]. Furthermore, health professionals’ mutual relationship with the person is essential to their understanding of “personhood” generally and individual people in particular [28].

The findings of the current study show that primary care workers face many organizational challenges such as understaffing and lack of qualified nurses, which led to heavy workloads and insufficient time to optimally care for elderly people with diabetes, and that important documentation and nursing care plans and patient profiles were not updated. Further, clinical care and service delivery were not routinely evaluated, and staff members had little time to update diabetes knowledge. These findings are similar to those of Morgan et al. [29] and suggest that workload issues such as inadequate staffing levels affect the ability to provide high-quality care in hospitals and nursing homes.

Another consequence of understaffing and the lack of qualified nurses is that unqualified personnel are given responsibilities they are not trained to provide, which can have adverse outcomes for community-based elderly people, including unnecessary hospital admissions. Previous studies identified the devalued image of nurses who work with elderly people. A systematic review of the experiences of nurses as managers and leaders in aged care suggests there is a need for specific education focused on clinical leadership and opportunities for professional development [30]. Geriatric nursing is a specialized and complex area of health care and organizational barriers often prevent continuing education and skills development.

## Conclusion and implications for clinicians and policy-makers

Professional networks that encourage cooperation and acceptance of responsibility enable the members of small communities to build consensus and to identify common ground for action. Such communities of learning expand learning, develop skills, support members to gain experience, share tools, and develop both individual and organizational expertise. In the future, efforts should focus on establishing systems such that specialist health care contributes to developing expertise in a reinforced municipal health service in primary care settings in Norway. Specialist health care services and general practitioners with clinical expertise in managing diabetes can have a pivotal role in such education and reflective practice forums.
